# Perspective: Pragmatic Exercise Recommendations for Older Adults: The Case for Emphasizing Resistance Training

**DOI:** 10.3389/fphys.2020.00799

**Published:** 2020-07-03

**Authors:** Dallin Tavoian, David W. Russ, Leslie A. Consitt, Brian C. Clark

**Affiliations:** ^1^Ohio Musculoskeletal and Neurological Institute, Ohio University, Athens, OH, United States; ^2^School of Physical Therapy and Rehabilitation Sciences, University of South Florida, Tampa, FL, United States; ^3^Department of Biomedical Sciences, Ohio University, Athens, OH, United States; ^4^Diabetes Institute, Ohio University, Athens, OH, United States; ^5^Division of Geriatric Medicine, Ohio University, Athens, OH, United States

**Keywords:** aerobic, resistance, exercise, muscle, older adults, strength, cardiovascular, diabetes

## Abstract

Optimal health benefits from exercise are achieved by meeting both aerobic and muscle strengthening guidelines, however, most older adults (OAs) do not exercise and the majority of those who do only perform one type of exercise. A pragmatic solution to this problem may be emphasizing a single exercise strategy that maximizes health benefits. The loss of muscle mass and strength at an accelerated rate are hallmarks of aging that, without intervention, eventually lead to physical disability and loss of independence. Additionally, OAs are at risk of developing several chronic diseases. As such, participating in activities that can maintain or increase muscle mass and strength, as well as decrease chronic disease risk, is essential for healthy aging. Unfortunately, there is a widely held belief that adaptations to aerobic and resistance exercise are independent of each other, requiring the participation of both types of exercise to achieve optimal health. However, we argue that this assertion is incorrect, and we discuss crossover adaptations of both aerobic and resistance exercise. Aerobic exercise can increase muscle mass and strength, though not consistently and may be limited to exercise that overloads a particular muscle group, such as stationary bicycling. In contrast, resistance exercise is effective at maintaining muscle health with increasing age, and also has significant effects on cardiovascular disease (CVD) risk factors, type 2 diabetes (T2D), cancer, and mortality. We posit that resistance exercise is the most effective standalone exercise strategy for improving overall health in OAs and should be emphasized in future guidelines.

## Introduction

Over the next 40 years the number of adults over 65 years of age will more than double in the United States from 46 million to 98 million (Mather et al., 2015). In this context, the importance of habitual exercise as it relates to healthy aging cannot be overstated. For instance, there is overwhelming evidence that lifelong exercise can delay the onset of at least 40 chronic conditions/diseases (Ruegsegger and Booth, 2018). OAs are more likely to suffer from multiple chronic conditions and poor health status than young (18–44 years) or middle-aged (45–64 years) adults (National Center for Health Statistics, 2017) accompanied by an increasing rate of health care expenditures (Alemayehu and Warner, 2004). However, from a health economics perspective, OAs who participate in community exercise programs at least once per week have annual healthcare costs 21% lower than those who do not participate (Ackermann et al., 2003) due, at least in part, to the prevention or delay of chronic diseases (Ruegsegger and Booth, 2018).

**FIGURE 1:**
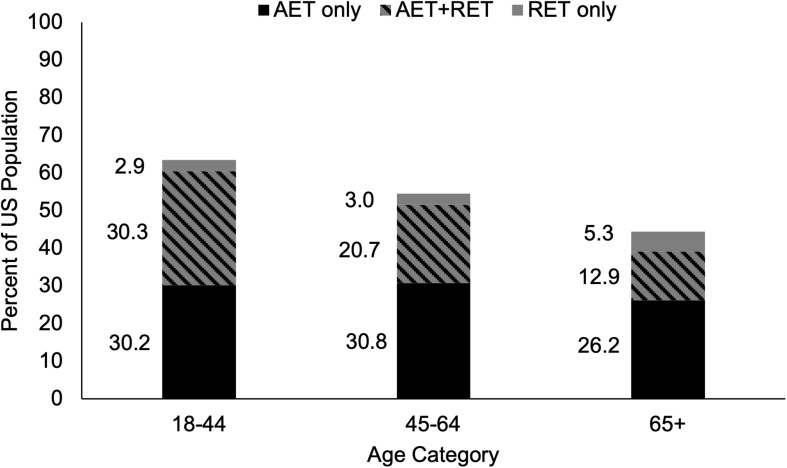
Proportion of Adults in the United States Meeting the 2008 Physical Activity Guidelines for Americans. The proportion of young, middle-aged, and older adults who self-report meeting only the aerobic exercise guidelines of 150 min per week of moderate-intensity or 75 min per week of vigorous-intensity aerobic exercise, only the muscle strengthening guidelines of activities that strengthen the major muscle groups at least two times per week, or both aerobic and muscle strengthening guidelines concurrently. As age increases, self-reported adherence to exercise guidelines declines. A greater proportion of adults meet aerobic exercise guidelines than muscle strengthening guidelines in all age categories. AET, aerobic exercise training; RET, resistance exercise training. Adapted from public-use data provided by National Center for Health Statistics (2018).

Nationally endorsed physical activity guidelines recommend a minimum of 150 min per week of Aerobic Exercise Training (AET), accompanied by muscle strengthening activities at least 2 days per week in order to maximize health benefits (US Department of Health Human Services, 2018). Unfortunately, only 13% of OAs achieve optimal health benefits by meeting both guidelines concurrently, whereas a third of OAs meet only AET or only muscle strengthening guidelines (National Center for Health Statistics, 2018; Figure 1). However, OAs may be overestimating their time spent in moderate-vigorous AET and/or misclassifying light-intensity activity as moderate-vigorous. For example, using accelerometer data it was reported that only 2.4% of OAs met the AET guidelines (Troiano et al., 2008), while another study reported that only 35% of OAs who self-reported meeting AET guidelines actually met them (Visser et al., 2014). Misclassification of intensity during muscle strengthening exercise is also a risk, however, this can be circumvented if exercises are performed to failure, even at low intensities (e.g., 30% of 1RM) (Mitchell et al., 2012; Van Roie et al., 2013). With the vast majority of OAs not exercising regularly or only performing one type of exercise, a more pragmatic approach of emphasizing a single exercise type may be warranted. Resistance Exercise Training (RET), arguably the most common muscle strengthening exercise, may be the most effective standalone exercise strategy for OAs as it can counteract the age-related loss of muscle mass, strength, and power that lead to poor physical function and loss of independence (Miszko et al., 2003; Liu and Latham, 2009). Additionally, there is evidence that RET can reduce the prevalence of T2D, cancer, CVD, and all-cause mortality (Stamatakis et al., 2018; Mcleod et al., 2019), historically believed to be achieved with AET. Emphasizing the importance of RET for OAs to physicians and policymakers is the first step to widespread acceptance of RET as an essential tool to combat age-related declines in health and physical function. In this Perspective article we will discuss recent evidence indicating that there are some potential crossover benefits for both types of exercise. Additionally, we will make the case that RET is the most effective standalone exercise strategy at improving overall health and reducing physical disability in OAs, and as such should be emphasized in future exercise guidelines.

## Physical Activity and Chronic Conditions in Older Adults

In 2008 the first nationally endorsed guidelines for physical activity were published based primarily on the Physical Activity Guidelines Advisory Committee’s Scientific Report, with the intention to be updated every 10 years (US Department of Health Human Services, 2008; US Department of Health Human Services, 2018; Physical Activity Guidelines Advisory Committee, 2018). Physical activity guidelines are essentially the same for OAs and adults under the age of 65, with the additional recommendation that OAs complete some sort of balance training as part of their weekly physical activity (US Department of Health Human Services, 2018). The 2018 national guidelines are intended as a resource for policy makers and health professionals, as well as the general public, for understanding the health benefits of different types, amounts, and intensities of physical activities for individual, community, and/or national implementation strategies (US Department of Health Human Services, 2018). The overarching goal is to increase the amount of physical activity for all age groups, in turn reducing the burden of lifestyle-induced diseases and conditions that are largely preventable. Current recommendations are based on the assumption that not all health benefits can be achieved through a single type of exercise, though greater emphasis is placed on the potential benefits of AET over muscle strengthening exercise (Physical Activity Guidelines Advisory Committee, 2018). Given the evidence presented herein, these traditional beliefs should be revisited.

As previously mentioned, OAs are at higher risk of accumulating chronic health conditions (National Center for Health Statistics, 2017). Additionally, OAs are at risk of developing sarcopenia, a progressive muscular condition characterized by low muscle mass and strength (Cruz-Jentoft et al., 2019) which contributes to the loss of functional independence (Trombetti et al., 2016). It is estimated that muscle mass decreases by 10% by age 50, and that nearly half of muscle mass is lost by age 80 (Lexell et al., 1988) whereas knee extensor strength and power decline by 10–15% per decade after the age of 50 (Lindle et al., 1997; Liu and Fielding, 2011). Additionally, it has been reported that 10–15% of OAs suffer from chronic and disabling conditions that result in irreversible frailty (Vellas and Sourdet, 2017). Importantly, an individual can gain the health benefits of physical activity, regardless of age, as long as the threshold for irreversible frailty has not been reached (Serra-Rexach et al., 2011; Cadore et al., 2014). Beginning regular exercise later in life results in lower incidence of physical and/or cognitive limitations, major chronic diseases, and poor mental health compared to those who remain inactive (Hamer et al., 2014) and improvements have even been seen in previously sedentary nonagenarians (Serra-Rexach et al., 2011; Cadore et al., 2014).

## Aerobic Exercise Training

There are several types of activity that are classified as AET, including, but not restricted to, running, hiking or brisk walking, dancing, swimming, aerobic classes or water aerobics, bicycle riding, and yard work, such as raking or pushing a lawn mower (US Department of Health Human Services, 2018). AET is known to reduce all-cause and CVD-mortality in a curvilinear dose-response manner, wherein the slope of the curve is steepest early in the relationship, but levels off with increased volume (Hamer and Chida, 2008; Kelly et al., 2014). For example, Sadarangani et al. (2014) reported decreased risk of all-cause and CVD mortality of 35% and 40%, respectively, in adults with diabetes who were meeting or exceeding the aerobic guidelines, compared to those who did not exercise. However, they also reported that those who performed some AET, but did not meet the AET guidelines, still had 26% and 32% lower all-cause and CVD mortality risk, respectively (Sadarangani et al., 2014), implying that some AET is better than none, and nearly as good as meeting the guidelines. Other well-known benefits include lower incidence of CVD (Wahid et al., 2016), reduced blood pressure (Cornelissen and Smart, 2013), improved Cardiorespiratory Fitness (CRF) (Lin et al., 2015), and lower incidence of T2D and risk of cancer (Kyu et al., 2016; Wahid et al., 2016). Attenuation of long-term weight gain has also been reported (Moholdt et al., 2014), though short-term weight loss is minimal without the addition of caloric restriction (Franz et al., 2007). There is also moderate evidence that AET can reduce fall risk (Gillespie et al., 2012), increase bone mineral density (Benedetti et al., 2018), and delay onset and progression of physical disability in OAs (Tak et al., 2013). While it is clear that AET can affect several aspects of human health, the inclusion of muscle strengthening activities in the guidelines implies that AET does not increase muscular strength. However, several investigations in OAs have reported increased strength in response to AET (Izquierdo et al., 2004; Harber et al., 2009; Lovell et al., 2010; Hollings et al., 2017; Timmons et al., 2018), though careful review of the methodology suggests these adaptations may depend on the mode of exercise employed.

The rhythmic and continuous nature of AET requires that muscular contractions are of low enough intensity that they can be repeated for multiple cycles in order to reach adequate exercise duration (i.e., 30 min per day) (Chodzko-Zajko et al., 2009), and low-intensity muscle contractions (i.e., <60% of maximal) are typically less effective at increasing muscle mass if not performed to failure (Wernbom et al., 2007). Because AET has traditionally been conceived as exercise for the heart, measures of muscle mass, strength, power, and quality have largely been neglected. The few studies that have addressed muscular adaptations to AET report mixed results (Grgic et al., 2019). A recent meta-analysis comparing the hypertrophic response to either AET or RET found that while some AET protocols can result in knee extensor hypertrophy, RET is more effective at both the whole-muscle and myofiber level (Grgic et al., 2019). Of the included studies, none that utilized walking or running exercise resulted in hypertrophy, wherein half of those that utilized stationary bicycling resulted in hypertrophy, though not to the same extent as RET (Grgic et al., 2019). A number of other studies without a RET comparison group have reported hypertrophic effects using stationary bicycling in both young and OAs (Harber et al., 2009, 2012; Konopka et al., 2010, 2014; Konopka and Harber, 2014). However, muscle size is, at best, a modest contributor to strength changes with exercise in OAs (Lee et al., 2019), and few studies have measured changes in strength in response to AET.

Increases in knee extensor isometric force and/or 1RM squat have been reported in the range of 11–35% after 12–16 weeks of stationary bicycling in OAs (Izquierdo et al., 2004; Harber et al., 2009; Lovell et al., 2010), occasionally increasing to a greater extent than time-matched RET (Timmons et al., 2018). However, walking exercise does not appear to increase knee extensor strength (Rooks et al., 1997; Kubo et al., 2007; Ozaki et al., 2011). Pooling different modes of AET results in large heterogeneity, exemplified by a meta-analysis in adults with coronary heart disease reporting changes in lower body strength ranging from −15.8% to +22.0% (median +6.3%) (Hollings et al., 2017). However, while the effects of AET on strength gains are inconsistent, long-term AET may at least protect muscular strength from age-related declines, as OAs who regularly participate in AET (10+ years) have higher knee extensor strength than sedentary OAs (Crane et al., 2013). Measurement of muscle power and quality is rare in AET studies, but some improvements have been reported (Harber et al., 2009; Konopka et al., 2010; Brightwell et al., 2019). From the data summarized herein it appears that both muscle mass and strength can be improved with AET, particularly in OAs, but this has only consistently been demonstrated in response to stationary bicycling. It should also be noted that AET is unlikely to have a global effect on muscle strength and mass, as improvements are specific to the muscles being used (i.e., lower extremities) (Physical Activity Guidelines Advisory Committee, 2018).

## Resistance Exercise Training

It is widely accepted that RET promotes hypertrophy and strength gains at all ages (Peterson et al., 2010, 2011; Steib et al., 2010; Churchward-Venne et al., 2015; Law et al., 2016), and that muscular power can be increased when a high-velocity component is included in the RET protocol (Bean et al., 2009). Similar to AET, there appears to be a dose-response relationship regarding RET and health benefits, wherein higher intensities and higher volumes of RET result in greater improvements in strength and mass in OAs (Peterson et al., 2010, 2011; Steib et al., 2010; Churchward-Venne et al., 2015; Law et al., 2016). Whether the adaptive response to RET is equivalent in both young and old adults is still debatable, as several investigations have reported no difference between age groups (Häkkinen et al., 1998; Roth et al., 2001; Newton et al., 2002; Walker and Häkkinen, 2014), while others have reported a blunted response in OAs (Raue et al., 1985; Lemmer et al., 2000; Macaluso et al., 2000; Martel et al., 2006). Regardless, RET is clearly beneficial for musculoskeletal health, and is likely the most effective strategy for maintaining and/or increasing muscle mass and strength with age (Law et al., 2016) in turn preventing and potentially reversing sarcopenia and delaying loss of independence (Evans, 1996). Muscle quality, fatigue resistance, and physical function are also improved with RET (Hunter et al., 2004). Additionally, bone mineral density increases (Going and Laudermilk, 2009) and blood pressure is reduced to an equal or greater extent with RET compared to AET (MacDonald et al., 2016).

The benefits of RET, as summarized in the 2018 Scientific Report, include (1) reductions in blood pressure equivalent to AET, (2) improved physical function, (3) reduced risk of falls and injury due to falls, and (4) maintenance of lean body mass during a program of weight (Physical Activity Guidelines Advisory Committee, 2018). The effects of muscle strengthening exercise on all-cause mortality, CVD mortality, CVD risk, T2D risk, and cancer risk were not addressed in the report (Physical Activity Guidelines Advisory Committee, 2018) but not due to lack of available data. A recent analysis of nearly 400,000 Americans (age range 18–80) reported that individuals meeting muscle strengthening guidelines alone had lower prevalence of hypertension, hypercholesterolemia, diabetes, myocardial infarction, and heart disease than those only meeting AET guidelines (Bennie et al., 2019). Additionally, regular RET is associated with reduced all-cause mortality (Stamatakis et al., 2018; Liu et al., 2019), cancer incidence (Keum et al., 2016) and mortality (Stamatakis et al., 2018), CVD morbidity and number of CVD events (Liu et al., 2019), and T2D risk and markers of metabolic dysregulation (i.e., glucose disequilibrium and insulin resistance) (Grøntved et al., 2012), independent of AET participation. A prospective cohort study from the Health Professionals Follow-up Study reported a comparable dose-response relationship between increased time spent on RET or AET and lower risk of T2D in men (Grøntved et al., 2012). Causation can only be inferred from these reports, though there have been numerous randomized control trials and epidemiological studies that have attempted to better define these associations, which are discussed in the next paragraph (Smutok et al., 1993; Tanasescu et al., 2002; Gelecek et al., 2012; Grøntved et al., 2012; Yang et al., 2014; MacDonald et al., 2016; Hollings et al., 2017; Shiroma et al., 2017; Villareal et al., 2017; Ihalainen et al., 2019).

As previously mentioned, RET significantly reduces blood pressure, particularly in hypertensive individuals (MacDonald et al., 2016). The effect of RET on additional CVD and/or T2D risk factors has been reported as similar to those observed in response to AET. AET was once thought to be the lone type of exercise to reduce body fat and insulin resistance, however, research now supports RET as an effective treatment, especially if prescribed at moderate volumes and/or frequency (Villareal et al., 2017; Ihalainen et al., 2019). For example, Ihalainen et al. (2019) reported significant reductions in fat mass when RET was performed 3 days per week for 6 months, but not when performed one or 2 days per week. Additionally, RET appears to be more effective than AET at reducing fat mass (−7.3 kg vs. −6.3 kg) and attenuating loss of thigh muscle volume (−1.9% vs. −6.2%) when combined with caloric restriction (Villareal et al., 2017). OAs with T2D can also improve skeletal muscle insulin action in response to RET, independent of changes in skeletal muscle mass (Holten et al., 2004), highlighting the multifaceted benefits of RET in the skeletal muscle of OAs. Regarding CRF, RET produces similar improvements as AET (15.6% and 20.1%, respectively) in OAs with coronary heart disease (Hollings et al., 2017). Additionally, a review by Ozaki et al. (2013) reported that CRF consistently increased in response to RET in OAs (6 of 9 included studies), while improvements were rare in young adults (3 of 17 included studies). The reason for a greater response in OAs is unclear, but may be related to the lower baseline levels of CRF associated with increasing age (Jackson et al., 2009). Additional risk factors of CVD and/or T2D that reportedly improve in response to RET in adults include insulin sensitivity (Ibañez et al., 2005), lipids and lipoproteins (Kelley and Kelley, 2009; Yang et al., 2014), triglycerides (Kelley and Kelley, 2009; Yang et al., 2014), and glycosylated hemoglobin (Gordon et al., 2009). Taken together, these results underline the critical role that RET may have in preventing and treating detrimental health conditions that target OAs.

## Emphasizing RET for Older Adults

**FIGURE 2:**
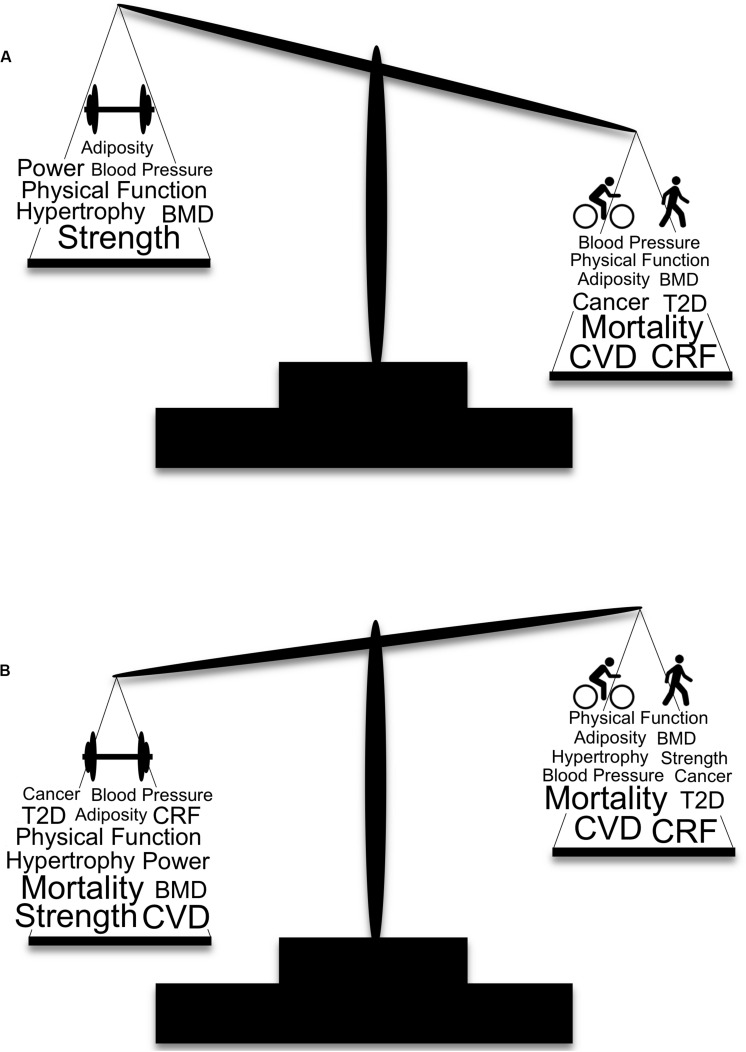
Traditional and modern depictions of the weighted importance of aerobic and muscle strengthening exercise and selected health benefits. **(A)** The traditional view of physical activity and health is based on the tenet that adaptations from AET and RET are largely independent of one another, with recommendations for AET given more weight for their beneficial effects on cardiovascular disease and mortality. **(B)** Our modern view of physical activity and health that includes the crossover benefits of AET and RET, indicating greater weight should be given to RET over AET. Larger font indicates a greater effect of the specific health benefit. BMD, bone mineral density; CRF, cardiorespiratory fitness; CVD, cardiovascular disease; T2D, type 2 diabetes.

Muscle strengthening activities result in beneficial musculoskeletal adaptations that are not consistently seen in response to AET, which is the primary reason for their inclusion in the current guidelines for the general population (US Department of Health Human Services, 2018). It is our belief that the emphasis of muscle strengthening exercise is even more critical for OAs because of the higher risk of sarcopenia and loss of independence in this population (Yazar and Yazar, 2019). There are several barriers to physical activity in OAs that can be targeted to increase participation [e.g., time, cost, disinterest, ongoing pain or illness, fear of injury, and feeling too old (Rasinaho et al., 2007; Aily et al., 2017; Burton et al., 2017a)]. Additional barriers specific to RET include perceived complexity of RET programs, lack of knowledge, and lack of age-appropriate programs (Phillips and Winett, 2010; Burton et al., 2017a, b). Addressing these barriers will be a necessary step in achieving widespread adherence to muscle strengthening guidelines. In contrast, the guidelines for AET are simplistic and can be met without specialized equipment or training (e.g., walking), though walking activity is affected by seasonal changes (Kimura et al., 2015). Presumably this is linked to why a greater proportion of OAs in the United States report meeting the AET guidelines than the muscle strengthening guidelines [39% and 18%, respectively (Figure 1; National Center for Health Statistics, 2018)]. Interestingly, 71% of OAs who report meeting muscle strengthening guidelines also meet the AET guidelines, whereas only 33% of OAs who report meeting AET guidelines also meet muscle strengthening guidelines (National Center for Health Statistics, 2018) suggesting that emphasizing muscle strengthening activities for OAs may indirectly result in greater AET participation. Considering the fact that 87% of OAs report either not exercising regularly or only meeting the guidelines for one type of exercise (National Center for Health Statistics, 2018) implementing a new approach to increasing exercise participation is necessary. Additionally, self-isolation due to the ongoing COVID-19 pandemic likely has detrimental effects on physical activity (Peçanha et al., 2020), making this a critical time to leverage at-home exercise programs (Hammami et al., 2020). Specifically, the creation of simple, age-appropriate, and educational RET programs that are easily accessible would address existing barriers to widespread adherence, protect OAs from exposure by not requiring gym attendance, and reduce sedentary behavior associated with self-isolation. Promoting RET as the exercise type with the greatest overall effect on health is a reasonable strategy, particularly for OAs. Of primary importance is overcoming the widespread belief that the benefits of AET and RET are independent of one another (Figure 2).

## Conclusion

It is the opinion of the authors that optimal benefits from exercise are achieved by meeting both AET and muscle strengthening guidelines concurrently, and that the Physical Activity Guidelines for Americans are rational and efficacious. However, given the fact that the majority of OAs either do not exercise regularly or only perform one type of exercise, a pragmatic approach of emphasizing the exercise strategy with the greatest overall effect is now warranted. OAs have a higher prevalence of CVD, cancer, osteoporosis, and T2D compared to young or middle-aged adults, highlighting the importance of participating in activities that can reduce the risk of developing these conditions. Furthermore, OAs are at risk of losing muscle mass and strength at an accelerated rate, increasing their risk of developing the aforementioned conditions, as well as loss of independence and mortality. Therefore, participating in activities that increase, or at a minimum, maintain, muscle mass and strength should be a critical component of exercise prescription for OAs. The historical belief that the benefits of AET and RET are independent of one another, with minimal crossover, is no longer founded. However, despite the potential for some types of AET (i.e., stationary bicycling) to have an impact on muscle strength and mass in targeted muscles, RET remains the most consistent and effective method for global muscular adaptations. In addition, given the mounting evidence that RET is just as beneficial as AET at mitigating chronic diseases highly prevalent in the aging population, we posit that, as a standalone exercise strategy, RET has the greatest effect on overall health in OAs and should be emphasized in future guidelines, particularly as an entry-level program for non-exercisers.

## Author Contributions

DT and BC conceived of the manuscript. DT wrote the initial draft of the manuscript. BC, DR, and LC critically reviewed the manuscript. All authors contributed to the refinement of the final manuscript.

## Conflict of Interest

In the past 5-years, BC has received research funding from NMD Pharma, Regeneron Pharmaceuticals, Astellas Pharma Global Development, Inc., and RTI Health Solutions for contracted studies that involved aging and muscle related research. In the past 5-years, BC has received consulting fees from Regeneron Pharmaceuticals, Zev Industries, and the Gerson Lehrman Group for consultation specific to age-related muscle weakness. BC is a co-founder with equity of AEIOU Scientific. The remaining authors declare that the research was conducted in the absence of any commercial or financial relationships that could be construed as a potential conflict of interest.

## Abbreviations

1RM: one repetition maximum
AET: aerobic exercise training
CRF: cardiorespiratory fitness
CVD: cardiovascular disease
OAs: older adults
RET: resistance exercise training
T2D: type 2 diabetes.
